# Revision of the genus *Paralipsis* Foerster, 1863 (Hymenoptera, Braconidae), with the description of two new species

**DOI:** 10.3897/zookeys.606.9656

**Published:** 2016-07-21

**Authors:** Cornelis van Achterberg, Nilo F. Ortiz de Zugasti Carrón

**Affiliations:** 1 Department of Terrestrial Zoology, Naturalis Biodiversity Center, Postbus 9517, 2300 RA Leiden, The Netherlands; 2 Faculty of Medicine, Katholieke Universiteit Leuven, Herestraat 49, 3000 Leuven, Belgium

**Keywords:** Paralipsis, Aphidiinae, new species, Spain, Netherlands, Germany, key, biology, endoparasitoid, social parasite, root aphids, ants, Lasius grandis

## Abstract

The Palaearctic genus *Paralipsis* Foerster, 1863 (Hymenoptera: Braconidae: Aphidiinae) is revised and two new species are described: *Paralipsis tibiator* van Achterberg & Ortiz de Zugasti, **sp. n.** from Spain and *Paralipsis planus* van Achterberg, **sp. n.** from the Netherlands. Some biological notes are supplied for *Paralipsis tibiator* **sp. n.** A key to the four known species is added and all species are illustrated.

## Introduction

The subfamily Aphidiinae (Hymenoptera: Braconidae) contains exclusively koinobiont parasitoids of ovoviviparous aphids (Aphididae sensu lato) (Yu et al. 2012; Shaw and Huddleston 1991). Few genera specialise on concealed hosts and *Paralipsis* Foerster, 1863, is one of them, by attacking root aphids associated with ants. The genus is restricted to the Palearctic region and only two valid species are known: *Paralipsis enervis* (Nees, 1834) (West Palaearctic) and *Paralipsis eikoae* (Yasumatsu, 1951) (East Palaearctic) (Yu et al. 2012). The detection of a new species of the genus in Spain by the second author triggered a revision of the genus and resulted in the discovery of a second new species from the Netherlands.

## Material and methods

The second author detected the first *Paralipsis tibiator* sp. n. female during a routine myrmecological survey and conserved it in 70% ethanol. The following year, a focused search was undertaken to collect more *Paralipsis* by nest excavation and aspiration of the parasitoids. In addition, during two days, at haphazard moments, short (approx. 15 minutes) observations were conducted totalling about three hours. The specimens of *Paralipsis planus* sp. n. and *Paralipsis enervis* (Nees) were collected either in Malaise traps or in pit-fall traps and conserved in 70% ethanol. The specimens were prepared using the AXA method (van Achterberg 2009; van Achterberg et al. 2010) and glued on card points or pinned on minutins. Observations and descriptions were made with an Olympus SZX11 stereomicroscope and fluorescent lamps. Photographic images were made with an Olympus motorized stereomicroscope SZX12 and processed with Adobe Photoshop CS5, mostly to adjust the size and background. The examined material is deposited in collection of the Naturalis Biodiversity Center
(RMNH), Leiden. POL stands for the distance between both posterior ocelli and OOL for the distance between posterior ocellus and compound eye.

### Biology

Ants constitute complex and well organized societies, which normally defence their nests viciously against intruders (Hölldobler and Wilson 1990). Nonetheless, several arthropods belonging to the class Arachnida or to main orders as Hymenoptera, Orthoptera and Coleoptera, have evolved to overcome this defence. As a result, symbiotic relationships ranging from mutualism, parasitism and commensalism to inquilinism occur in ant nests (Kistner 1982, Völkl et al. 1996). The case of the myrmecophilic aphidiine parasitoid wasp genus *Paralipsis* is more complicated, because they parasitise root aphids herded by ants (Takada and Hashimoto 1985). Once the parasitoid wasp infiltrates the ant colony, it finds a source of food (aphids) for its offspring protected by ants and thus sheltered from potential predators.

On 23 July 2014 two small wasps were seen in a nest of Lasius (Lasius) grandis Forel, 1909, located under a small rock at the foot of a *Cedrus* sp. (cedar) with abundant grass cover (lawn, Poaceae) at the Parque del Oeste (Madrid, Spain; 40°25'55.8"N, 3°43'43.7"W). Both wasps were occupying the galleries jointly with the ants; one was collected and the other one escaped flying. On 3 July 2015 in the same park (40°26'05.5"N, 3°43'27"W) a *Lasius grandis* nest at the foot of a *Populus alba* tree (white poplar), also covered with abundant *Poaceae* turf, was excavated and two additional females were collected. The following root aphids were found in the nest: an adult female of *Tetraneura ulmi* (Linnaeus, 1758), a nymph of *Tetraneura nigriabdominalis* (Sasaki, 1899), a nymph of *Aploneura lentisci* (Passerini, 1856) and two nymphs of *Forda formicaria* (von Heyden, 1837).

One female wasp was kept alive for two days along with ten ant workers of the same nest where the wasp was found. They were kept in a plastic container (8 cm × 8 cm × 3 cm) with supply of moisture and fed once with diluted honey. During this period, the wasp actively looked for the company of the ants. Upon disturbing the artificial nest, the wasp was always, and promptly, looking for a concentration of standing (not running) ants to join. Most of the time the wasp was hiding under the legs of the ants and sometimes walking around the group. The wasp was observed being frequently groomed and antennated by the ants. The wasp always showed a submissive behaviour and it was once observed actively antennating an ant, an action that elicited ant-wasp trophallaxis. While the first specimen was kept alive along with the ants, no wasp-ant rubbing such as is described by Takada and Yashimoto (1985) was observed. Probably, the rubbing behaviour was not observed because the chemical mimicry was already obtained.

In an ongoing study on aphid-ant relationships at a similar environment in Spain, so far *Lasius grandis* has been observed attending only *Forda formicaria* root aphids (Pérez Hidalgo, pers. com.). During this study *Paralipsis tibiator* sp. n. has been observed parasitizing *Forda formicaria* aphids being attended by *Lasius grandis*. It suggests that *Forda formicaria* is the preferred host of *Paralipsis tibiator* sp. n., but we cannot rule out that other root aphids are chosen as hosts. *Lasius grandis* “is the most abundant species of the subgenus on the Iberian peninsula” (Seifert 1992) with a continuous distribution across the Iberian Peninsula (Gómez and Espadaler 2007). Also *Forda formicaria* occurs all over the Peninsula (Nieto Nafria et al. 2003). Hence, it can be expected that *Paralipsis tibiator* sp. n. occurs in nests of *Lasius grandis* across the Iberian Peninsula.

## Systematics

### Paralipsis

Taxon classificationAnimaliaHymenopteraBraconidae

Foerster, 1863

Paralipsis Foerster, 1863: 248, 250; Starý 1958: 85; Mackauer 1968: 22; Takada 1976: 1. Type species: Aphidius enervis Nees, 1834 (by original designation).
Myrmecobosca Maneval, 1940: 9. Type species: Myrmecobosca mandibularis Maneval, 1940 (by original designation). Synonymised with Paralipsis Foerster, 1863, by Starý (1958).

#### Diagnosis.

**Figures 1–7.**
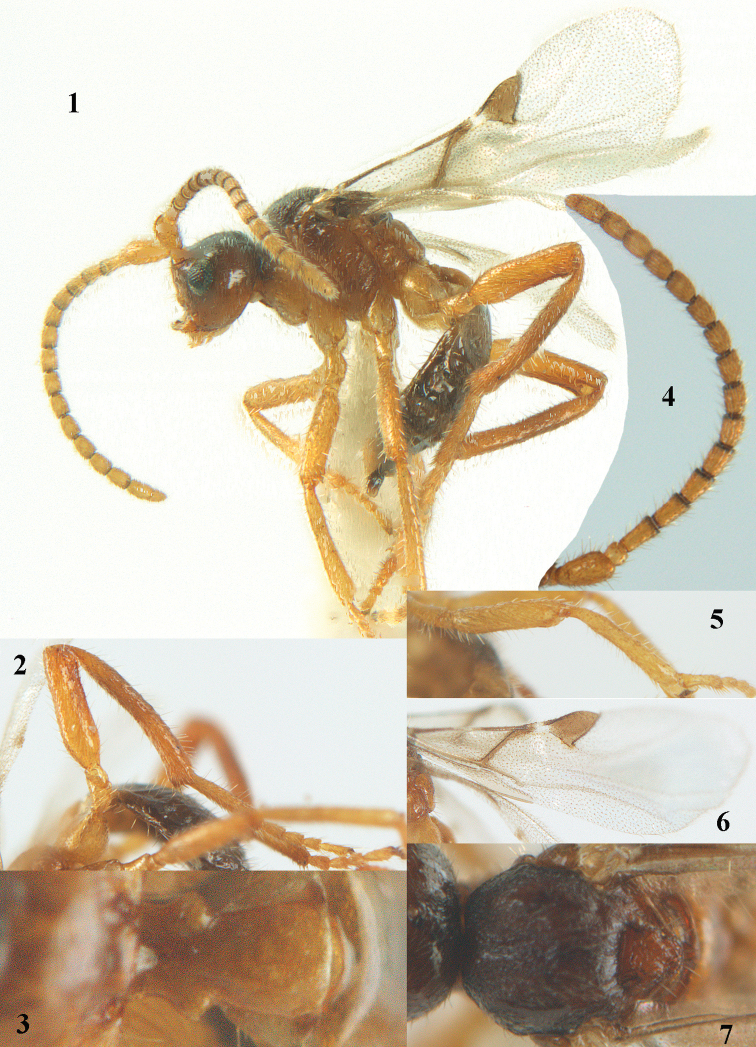
*Paralipsis eikoae* (Yasumatsu), female, Japan. **1** habitus lateral **2** hind leg **3** first metasomal tergite dorsal **4** antenna **5** fore leg **6** wings **7** mesonotum dorsal.

Veins r + SR and 1-R1 of fore wing absent and if weakly indicated then continuous with postero-basal border of pterostigma (Fig. 1); pterostigma conspicuous and wide triangular (Figs 1, 14, 25); scapus much larger than pedicellus and apically widened (Fig. 4); first tergite parallel-sided or weakly widened posteriorly (Figs 3, 11, 19, 27); labial palp with 1 segment and maxillary palp with 2 segments; scutellum protuberant, but of ♀ of *Paralipsis eikoae* and of males flattened; mandible strongly narrowed and twisted apically, with two minute apical teeth and with fine ventral carina (Fig. 22); fore and middle coxa nearly triangularly enlarged (Fig. 21); precoxal sulcus absent; ventrally head with long combined occipital and hypostomal carina (Fig. 23); length of body 2.0–2.7 mm.

**Figures 8–15.**
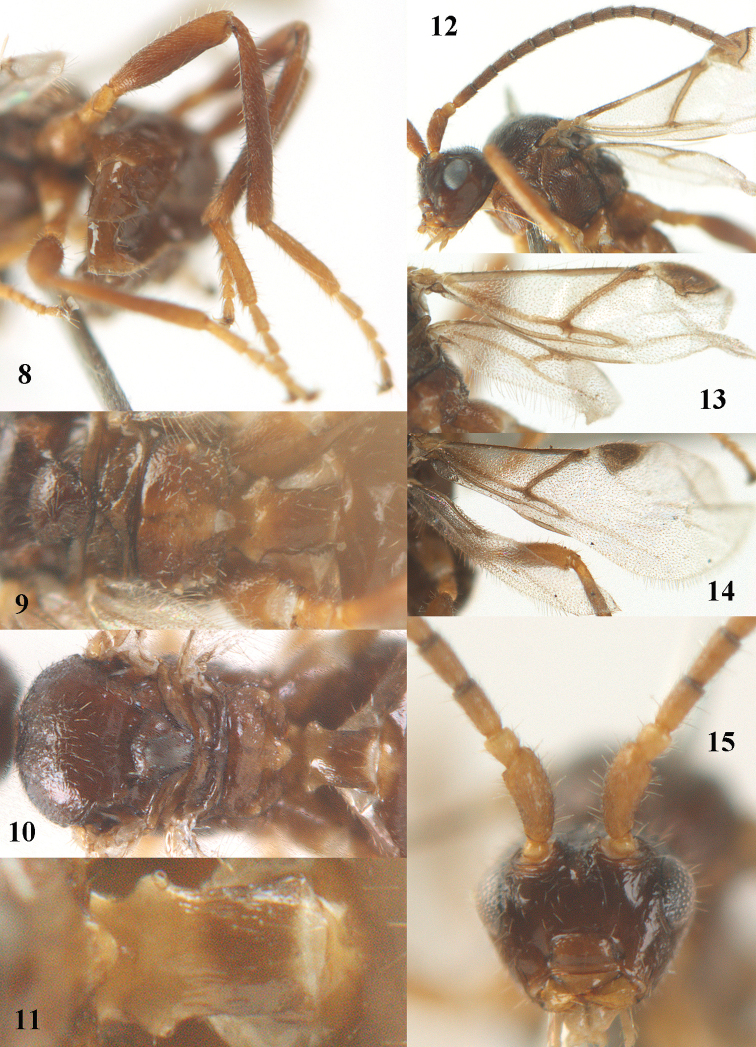
*Paralipsis enervis* (Nees), female, Netherlands, but **10–11, 14–15** Germany. **8** hind leg **9** posterior part of mesosoma and first metasomal tergite dorsal **10** mesosoma and first metasomal tergite dorsal **11** first metasomal tergite dorsal **12** antenna **13–14** wings **15** head anterior.

**Figures 16–20.**
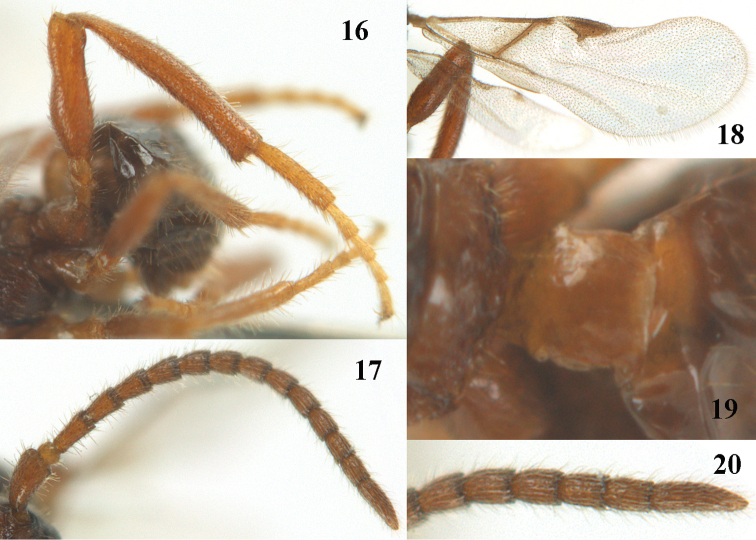
*Paralipsis planus* van Achterberg, sp. n., female, holotype. **16** hind leg **17** antenna **18** wings **19** first metasomal tergite dorsal **20** apex of antenna.

**Figures 21–27.**
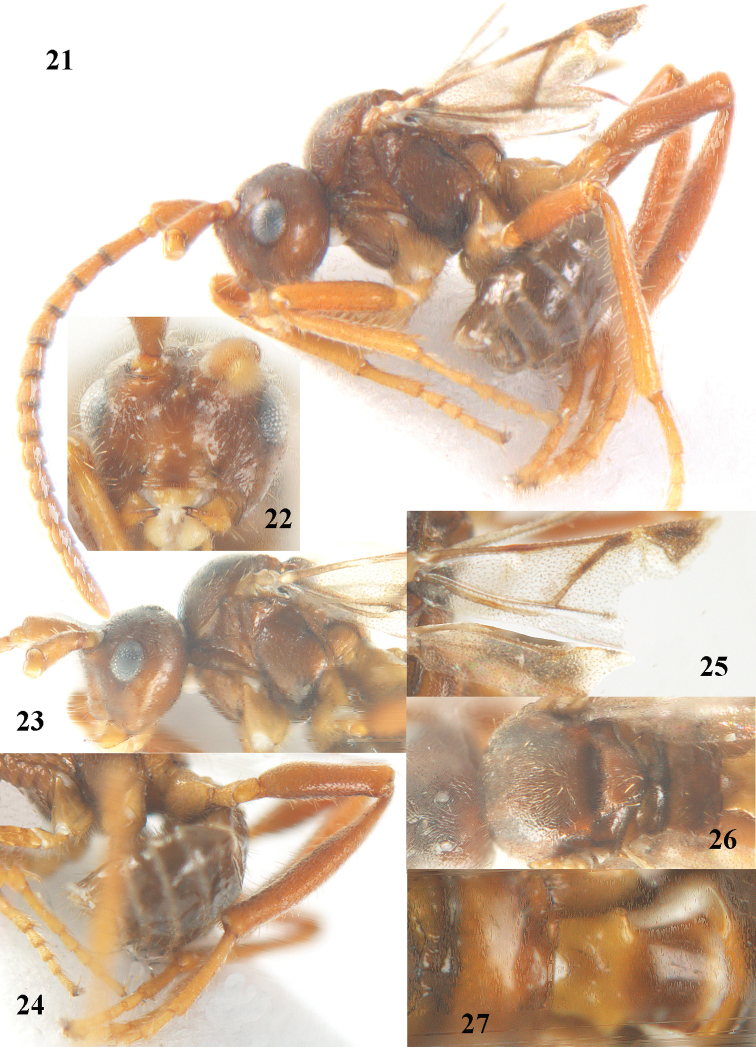
*Paralipsis tibiator* van Achterberg & Ortiz de Zugasti, sp. n, female, holotype. **21** habitus lateral **22** head anterior **23** head and mesosoma lateral **24** hind leg **25** wings **26** mesosoma dorsal **27** propodeum and first metasomal tergite dorsal.

#### Biology.

Myrmecophylic endoparasitoids of root aphids (Aphididae) (Maneval 1940; Hincks 1949, 1958; Starý 1958; Pontin 1960). Fore wing frequently mutilated, possibly by ants during their stay inside the ant nest.

#### Distribution.

Palaearctic; four species, of which two new to science.

#### Key to species of *Paralipsis* Foerster

**Table:** 

1	Secondfourth segments of fore tarsus about as long as wide in dorsal view and with medium-sized bristles apically (Figs 1, 8); hind tibia medially and femur subbasally parallel-sided (Figs 1, 2, 8); subapically scapus subparallel-sided in lateral view (Figs 1, 4, 12); [rhinaria absent on fifth antennal segment; pterostigma nearly straight baso-posteriorly (Figs 1, 6, 13, 14)]	**2**
–	Secondfourth segments of fore tarsus distinctly longer than wide in dorsal view and with long apical bristles (Figs 21, 24); hind tibia medially and femur subbasally widened (Figs 16, 24); scapus distinctly widened dorsally in lateral view (vase-shaped: Figs 17, 21); [vein 2-1A of fore wing obsolescent or absent (Fig. 18); middle antennal segments of ♀ narrowed basally (Figs 17, 21)]	**3**
2	Scutellum distinctly convex and shiny, smooth (Fig. 9); third–fifth antennal segments longer (Fig. 12); mesoscutum shiny and mainly smooth (Fig. 10); middle and hind femora and tibiae slenderer, hind tibia hardly or not sculptured (Fig. 8); vein 2-1A of fore wing largely or entirely sclerotized (Figs 13–14); first tergite less slender behind spiracles (Figs 9, 11); [width of first tergite at level of spiracles of ♀ equal to distance between spiracle and apex of tergite; middle antennal segments of ♀ more or less parallel-sided (Fig. 12)]; N & C. Europe	***Paralipsis enervis*** (Nees, 1834)
–	Scutellum irregularly flattened and dull, finely sculptured posteriorly (Fig. 7); third–fifth antennal segments rather short (Fig. 4); mesoscutum largely dull and distinctly finely sculptured (Fig. 7); middle and hind femora and tibiae more robust, hind tibia micro-sculptured (Figs 1-2); vein 2-1A of fore wing absent (Fig. 6); first tergite slender behind spiracles (Fig. 3); Japan (Honshu, Kyushu), Far East Russia	***Paralipsis eikoae*** (Yasumatsu, 1951)
3	Vertex and mesoscutum with satin sheen and vertex with dense short pubescence between sparse long setae (Fig. 26); first tergite more convex and less shiny, its maximum width at level of spiracles of ♀ 0.9 times distance between spiracle and apex of tergite (Fig. 27); mesopleuron with satin sheen (Fig. 23); antennal segments with adpressed setae (Fig. 21); fore basitarsus slender and with shorter setae (Fig. 21); hind basitarsus elongate (Fig. 24); pterostigma concave baso-posteriorly (Fig. 25); fifth antennal segment without distinct rhinaria; SW Europe	***Paralipsis tibiator* sp. n.**
–	Vertex and posteriorly mesoscutum shiny and vertex with sparse short pubescence between long setae; first tergite flat and shiny, its maximum width at level of spiracles of ♀ 0.7 times distance between spiracle and apex of tergite (Fig. 19); mesopleuron shiny; antennal segments with long erect setae (Figs 17, 20); fore basitarsus rather robust and with longer setae; hind basitarsus robust (Fig. 16); pterostigma straight baso-posteriorly (Fig. 18), but slightly concave in right wing of holotype; few rhinaria present on fifth antennal segment; NW Europe	***Paralipsis planus* sp. n.**

### Paralipsis eikoae

Taxon classificationAnimaliaHymenopteraBraconidae

(Yasumatsu, 1951)

Figs 1–7

Myrmecobosca eikoae Yasumatsu, 1951: 171–174.
Paralipsis eikoae ; Starý 1958: 89; Yasumatsu 1960: 57; Mackauer 1968: 22; Takada 1968: 91, 1976: 15.

#### Material.

1 ♀ (RMNH), “[**Japan**:] Mt. Hiei, 15.v.1996, H. Takada”, “Host: *Sappaphis piri*”, “*Paralipsis eikoae* (Yasumatsu), det. H. Takada, 2015”.

#### Diagnosis.

This species shares with *Paralipsis enervis* having the secondfourth segments of fore tarsus about as long as wide in dorsal view, the fore tarsus with medium-sized bristles apically (Figs 1, 8) and the hind tibia medially and femur subbasally parallel-sided (Figs 1, 2, 8). Differs by the dull and posteriorly finely sculptured scutellum (Fig. 7), the rather short third–fifth antennal segments (Fig. 4), the largely dull and distinctly finely sculptured mesoscutum (Fig. 7), the more robust middle and hind femora and tibiae, the micro-sculptured hind tibia (Figs 1–2), the vein 2-1A of fore wing absent (Fig. 6) and the slenderer first tergite (Fig. 3).

#### Biology.

Parasitoid of root aphids attended by the ants *Lasius sakagamii* Yamauchi & Hayashida, 1970 or *Lasius japonicus* Santschi, 1941 (Yu et al. 2012, Akino and Yamaoka 1998). Holotype male was collected from a nest of *Lasius japonicus* (published as *Lasius niger*; see Seifert 1992) in an old *Cryptomeria japonica* tree and the species was reared as parasitoid of the aphid *Sappaphis piri* Matsumura, 1918, on roots and subterranean stems of *Artemisia princeps* Pamp. (Takada 1976).

#### Distribution.

Reported from Japan and Far East Russia (Yu et al. 2012).

### Paralipsis enervis

Taxon classificationAnimaliaHymenopteraBraconidae

(Nees, 1834)

Figs 8–15

Aphidius enervis Nees, 1834: 26–27 (holotype male lost).
Paralipsis enervis ; Starý 1958: 85, 1961: 228–232; Hincks 1958: 20–21; Pontin 1960: 154–155; Mackauer 1968: 22.
Myrmecobosca mandibularis Maneval, 1940: 10–11. Synonymised with Paralipsis enervis (Nees, 1834) by Starý (1958).
Myrmecobosca linnei Hincks, 1949: 173–174. Synonymised with Paralipsis enervis (Nees, 1834) by Starý (1958).

#### Material.

1 ♀ (RMNH), “**Nederland**: Rotterdam (Z.H.), NS-driehoek”, “in ground-traps, 18.ix.1976, Insektenwerkgroep KNNV”; 1 ♀ (RMNH), id., but 20.viii.1976; 1 ♀ (RMNH), “Netherlands: Bennekom, 10.x.1971, D. Hille Ris Lambers”, “[ex] aphid mummy of *Brachycaudus jacobi* Stroyan”; 1 ♀ (RMNH), “[**Germany**:] German Dem. Rep., Museum Leiden”, “NSG Wernsdorfer See (n[ea]r Berlin), 1.vii.1979, G.N. Wendt”.

#### Diagnosis.

This species shares with *Paralipsis eikoae* having the second-fourth segments of fore tarsus about as long as wide in dorsal view, the fore tarsus with medium-sized bristles apically (Figs 1, 8) and the hind tibia medially and femur subbasally parallel-sided (Figs 1, 2, 8). Differs by the shiny and smooth scutellum (Fig. 9), the brownish scutellum, the longer third–fifth antennal segments (Fig. 12), the shiny and mainly smooth mesoscutum (Fig. 10), the slenderer middle and hind femora and tibiae, the hardly or not sculptured hind tibia (Fig. 8), the largely or entirely sclerotized vein 2-1A of fore wing (Figs 13–14) and the less slender first tergite (Figs 9, 11).

#### Biology.

Parasitoid of root aphids belonging to the genera *Anoecia*, *Anuraphis*, *Aphis*, *Brachycaudus*, *Chromaphis*, *Dysaphis*, *Forda*, *Geocia* and *Tetraneura* (Yu et al. 2012) and associated with *Lasius* ants. *Brachycaudus jacobi* Stroyan is a new host.

#### Distribution.

Reported from Andorra, Czech Republic, Finland, France, Georgia, Germany, Hungary, Kazakhstan, Macedonia, Moldova, Netherlands, Poland, Portugal, Romania, Slovakia, Spain, Sweden, UK and Serbia (Yu et al. 2012). The reports from Spain, Portugal and Andorra may concern *Paralipsis tibiator* sp. n.

### Paralipsis planus

Taxon classificationAnimaliaHymenopteraBraconidae

van Achterberg sp. n.

http://zoobank.org/C8E5EB50-4F3D-4071-9DE7-BFB816F31CE2

Figs 16–20

#### Material.

Holotype, ♀ (RMNH), “**Nederland**: Wijster (Dr.), opposite Biol. Stat., 28.vii.-14.viii.1972, C. v. Achterberg”.

#### Diagnosis.

Similar to *Paralipsis enervis* (Nees, 1834), but differs by the slenderer fore tarsus, the partly widened hind tibia and femur (Fig. 16) and the scapus distinctly widened dorsally in lateral view (vase-shaped: Fig. 17). Close to *Paralipsis tibiator* sp. n., but *Paralipsis planus* has the vertex and mesoscutum shiny and with sparse short pubescence between long setae, the first tergite flat and shiny, its maximum width at level of spiracles of ♀ 0.7 times distance between spiracle and apex of tergite (Fig. 19), the mesopleuron shiny, the apical antennal segments with long erect setae (Figs 17, 20), the fore basitarsus rather robust, the fifth antennal segment with few rhinaria and the hind basitarsus robust (Fig. 16).

Holotype, ♀, length of fore wing 2.0 mm, and of body 2.1 mm.

#### Description.

*Head*. Head 1.6 times wider than long medially in dorsal view and roundly narrowed behind eyes; antenna with 15 (left) or 16 (right) segments and 0.9 times as long as body, segments long erect setae (Figs 17, 20), third segment dull and 1.1 times as long as fourth segment, thirdfourth segments without rhinaria and widened apically and fifth segments with few rhinaria, third, fourth and penultimate (= 14^th^) segments 2.2, 1.8 and 1.4 times as long as wide, respectively; maxillary and labial palpi with 2 and 1 segments, respectively; length of maxillary palp 0.2 times height of head; distance between anterior tentorial pits 1.4 times distance between pit and eye; eye with rather long setae; face mainly smooth, convex ventrally and laterally rather sparsely setose, with setae directed downwards; clypeus distinctly convex and smooth, with few erect setae; frons nearly flat (except superficial impression in front of anterior ocellus), without median groove, shiny, punctulate and rather densely setose; vertex with sparse short pubescence between sparse long setae and temple roundly narrowed posteriorly and shiny; eye 0.9 times as long as temple in dorsal view; OOL:diameter of posterior ocellus:POL = 12:3:11; stemmaticum distinctly wider posteriorly than laterally; length of malar space 1.7 times basal width of mandible, malar depression absent.

*Mesosoma*. Length of mesosoma 1.3 times as long as high; pronotal side smooth and largely glabrous, with deep oblique groove and anteriorly short; mesopleuron mainly smooth, shiny, punctulate but superficially rugulose anteriorly and medially convex; pleural sulcus distinctly crenulate; metapleuron mainly rugose; mesoscutum with some micro-sculpture, posteriorly shiny and with dense short pubescence between long setae, but sparsely so posteriorly, antero-medially slightly depressed and with few striae; notauli absent on disc; scutellar sulcus very deep; scutellum strongly convex but slightly depressed antero-medially, posteriorly distinctly above level of mesoscutum, largely rugulose and with long setae; dorsal face of propodeum smooth and shiny, posterior face subvertical and indistinctly rugulose, without areolation and laterally with short setae.

*Wings*. Fore wing: pterostigma straight baso-posteriorly (Fig. 18), but slightly concave in right wing; pterostigma twice as long as wide and vein 1-R1 largely absent; first subdiscal cell open posteriorly and apically (Fig. 18), but veins 2-1A and CU1b as faintly pigmented and unsclerotized veins present.

*Legs*. Hind coxa mainly smooth, punctulate and setose; tarsal claws medium-sized and very slender; fore tarsal segments slender (secondfourth segments distinctly longer than wide in dorsal view), with long setae and with long apical bristles, but fore basitarsus rather robust; length of femur, tibia and basitarsus of hind leg 3.4, 6.1 and 4.8 times as long as wide, respectively; hind basitarsus robust (Fig. 16); hind femur subbasally and hind tibia medially widened (Fig. 16), both with erect setae; inner hind tibial spur 0.2 times as long as hind basitarsus.

*Metasoma*. First tergite smooth, flattened and shiny, its maximum width at level of spiracles of ♀ 0.7 times distance between spiracle and apex of tergite (Fig. 19), parallel-sided posteriorly, tergite 1.1 times long as wide apically; second tergite smooth and glabrous except some setae, third and following tergites smooth and only with a subapical row of long setae; length of visible (and sparsely setose) part of elliptical ovipositor sheath 0.05 times fore wing.

*Colour*. Head (including clypeus), mesosoma (but notaulic courses and posterior part of mesoscutum, scutellum, metanotum laterally and propodeum brown) and metasoma (but first tergite and second tergite basally brownish yellow) dark brown;, palpi, mandible, tegulae (but tegulum brown) and legs (but femora and tibiae brown and tarsi pale yellowish) brownish yellow; antenna brown, but pedicellus pale yellowish; ovipositor sheath pale brownish yellow, distinctly paler than tergites; pterostigma (but basally and apically pale yellowish) and veins mainly brown; wing membrane infuscate near vein 1-M of fore wing.

#### Biology.

Unknown.

#### Distribution.

Netherlands.

#### Etymology.

Named “planus” (Latin for “smooth, even”) because of the smooth and even first metasomal tergite.

### Paralipsis tibiator

Taxon classificationAnimaliaHymenopteraBraconidae

van Achterberg & Ortiz de Zugasti sp. n.

http://zoobank.org/9B89A20B-3950-4FAC-931C-8D1FDD2C9A85

Figs 21–27

#### Material.

Holotype, ♀ (RMNH), “**Spain**: Madrid, Parque del Oeste, from *Lasius grandis* nest, vii.2014, c. 600 m, N. Ortiz de Zugasti Carrón, RMNH”. Paratypes: 2 ♀ (RMNH), topotypic but 3.vii.2015.

#### Diagnosis.

Similar to *Paralipsis enervis* (Nees, 1834), but differs by the slenderer fore tarsus (Fig. 21), the partly widened hind tibia and femur (Fig. 24) and the scapus distinctly widened dorsally in lateral view (vase-shaped: Fig. 21). Close to *Paralipsis planus* sp. n., but *Paralipsis tibiator* has the vertex and mesoscutum with satin sheen and with dense short pubescence between sparse long setae (Fig. 26), the first tergite more convex and less shiny, its maximum width at level of spiracles of ♀ 0.9 times distance between spiracle and apex of tergite (Fig. 27), the mesopleuron with satin sheen (Fig. 23), the apical antennal segments with adpressed setae (Fig. 21), the fore basitarsus slenderer (Fig. 21), the fifth antennal segment without distinct rhinaria and the hind basitarsus elongate (Fig. 24).

Holotype, ♀, length of body 2.2 mm and of damaged fore wing 1.1 mm.

#### Description.

*Head*. Head 1.4 times wider than long medially in dorsal view and roundly narrowed behind eyes; antenna with 15 segments and as long as body, segments adpressed setose and setae rather short, third segment dull and 1.3 times as long as fourth segment, thirdfifth segments without rhinaria and widened apically, third, fourth and penultimate segments 2.2, 1.8 and 1.4 times as long as wide, respectively; maxillary and labial palp with 2 and 1 segments, respectively; length of maxillary palp 0.2 times height of head; distance between anterior tentorial pits 1.2 times distance between pit and eye (Fig. 22); eye with long setae; face mainly smooth, convex ventrally and laterally rather densely moderately setose, with setae directed downwards; clypeus distinctly convex and smooth, with long erect setae (Fig. 21); frons convex, with shallow median groove, rather dull, punctulate and densely setose; vertex with dense short pubescence between sparse long setae and temple roundly narrowed posteriorly and with satin sheen; eye 0.9 times as long as temple in dorsal view; OOL: diameter of posterior ocellus: POL = 3:1:3; stemmaticum distinctly wider posteriorly than laterally (Fig. 26); length of malar space 1.9 times basal width of mandible, malar depression absent.

*Mesosoma*. Length of mesosoma 1.2 times as long as high; pronotal side smooth and largely glabrous, anteriorly very short; mesopleuron mainly smooth, with satin sheen, punctulate and medially flattened; pleural sulcus mainly micro-crenulate; metapleuron with some micro-sculpture; mesoscutum with some micro-sculpture, with satin sheen and with dense short pubescence between sparse long setae, without medio-posterior groove; notauli absent on disc; scutellar sulcus very deep; scutellum strongly convex, far above level of mesoscutum (Fig. 23), largely smooth and setose; propodeum smooth and shiny, posterior face angled with dorsal face (Fig. 21), without areolation and laterally with few long setae.

*Wings*. Fore wing: pterostigma concave baso-posteriorly (Fig. 25); pterostigma twice as long as wide and vein 1-R1 largely absent; first subdiscal cell open posteriorly and apically (Fig. 21), vein 2-1A absent.

*Legs*. Hind coxa mainly smooth, punctulate and setose; tarsal claws medium-sized and very slender; fore tarsal segments slender (secondfourth segments distinctly longer than wide in dorsal view), with rather short setae and with long apical bristles (Fig. 21); length of femur, tibia and basitarsus of hind leg 3.6, 6.8 and 5.4 times as long as wide, respectively; hind femur subbasally and hind tibia medially widened (Fig. 24), both with erect setae; inner hind tibial spur 0.2 times as long as hind basitarsus.

*Metasoma*. First tergite smooth, rather convex and moderately shiny, its maximum width at level of spiracles of ♀ 0.9 times distance between spiracle and apex of tergite (Fig. 27), weakly diverging posteriorly, tergite 1.3 times as long as wide apically; second tergite smooth and setose, third and following tergites smooth and only with a subapical row of setae; length of visible (and sparsely setose) part of elliptical ovipositor sheath 0.05 times fore wing in paratype with complete wings.

*Colour*. Head (but clypeus brown), metasoma (but first tergite basally, narrowly apically and second tergite basally yellow) and mesoscutum (except brown notaulic courses) dark brown; antenna, palpi, mandible, tegulae, legs (but femora and tibiae brown) and propodeum brownish yellow; ovipositor sheath mainly dark brown, slightly paler than tergites; pterostigma (but basally and apically yellowish) and veins dark brown; wing membrane infuscate near veins and pterostigma.

*Variation*. Antenna of ♀ with 15 (3) segments; length of complete fore wing 1.8 mm and of body 2.2 mm; first tergite 1.3–1.5 times as long as wide apically; femora and tibiae brown or largely dark brown.

#### Biology.

Endoparasitoid of the aphid *Forda formicaria* (von Heyden, 1837) and a social parasite in nest of Lasius (Lasius) grandis Forel, 1909. The ant is known from the Iberian Peninsula, Maghreb, Balearic Islands, Macaronesia and SE France (http://antmaps.org/?mode=species&species=Lasius.grandis).

#### Distribution.

Spain.

#### Etymology.

Named “tibiator” (“tibia” is Latin for “shinbone”), because of the aberrant hind tibia.

## Supplementary Material

XML Treatment for Paralipsis

XML Treatment for Paralipsis eikoae

XML Treatment for Paralipsis enervis

XML Treatment for Paralipsis planus

XML Treatment for Paralipsis tibiator
